# ROHHAD and Prader-Willi syndrome (PWS): clinical and genetic comparison

**DOI:** 10.1186/s13023-018-0860-0

**Published:** 2018-07-20

**Authors:** Sarah F. Barclay, Casey M. Rand, Lisa Nguyen, Richard J. A. Wilson, Rachel Wevrick, William T. Gibson, N. Torben Bech-Hansen, Debra E. Weese-Mayer

**Affiliations:** 1 Department of Medical Genetics, Cumming School of Medicine, Alberta Children’s Hospital Research Institute, University of Calgary, Calgary, AB Canada; 2 Center for Autonomic Medicine in Pediatrics (CAMP) in Stanley Manne Children’s Research Institute and in Department of Pediatrics, Ann & Robert H. Lurie Children’s Hospital of Chicago, Chicago, IL USA; 3 Department of Physiology and Pharmacology, Alberta Children’s Hospital Research Institute and Hotchkiss Brain Institute, Cumming School of Medicine, University of Calgary, Calgary, AB Canada; 4 Department of Medical Genetics, University of Alberta, Edmonton, AB Canada; 5 Department of Medical Genetics, University of British Columbia and Child & Family Research Institute, Vancouver, BC Canada; 6 Pediatric Autonomic Medicine, Northwestern University Feinberg School of Medicine, Chicago, IL USA

**Keywords:** Pediatric obesity, Autonomic dysfunction, Hypothalamus, Hypoventilation, Genetics, Prader Willi syndrome

## Abstract

**Background:**

Rapid-onset obesity with hypothalamic dysfunction, hypoventilation, and autonomic dysregulation (ROHHAD) is a very rare and potentially fatal pediatric disorder, the cause of which is presently unknown. ROHHAD is often compared to Prader-Willi syndrome (PWS) because both share childhood obesity as one of their most prominent and recognizable signs, and because other symptoms such as hypoventilation and autonomic dysfunction are seen in both. These phenotypic similarities suggest they might be etiologically related conditions. We performed an in-depth clinical comparison of the phenotypes of ROHHAD and PWS and used NGS and Sanger sequencing to analyze the coding regions of genes in the PWS region among seven ROHHAD probands.

**Results:**

Detailed clinical comparison of ROHHAD and PWS patients revealed many important differences between the phenotypes. In particular, we highlight the fact that the areas of apparent overlap (childhood-onset obesity, hypoventilation, autonomic dysfunction) actually differ in fundamental ways, including different forms and severity of hypoventilation, different rates of obesity onset, and different manifestations of autonomic dysfunction. We did not detect any disease-causing mutations within PWS candidate genes in ROHHAD probands.

**Conclusions:**

ROHHAD and PWS are clinically distinct conditions, and do not share a genetic etiology. Our detailed clinical comparison and genetic analyses should assist physicians in timely distinction between the two disorders in obese children. Of particular importance, ROHHAD patients will have had a normal and healthy first year of life; something that is never seen in infants with PWS.

**Electronic supplementary material:**

The online version of this article (10.1186/s13023-018-0860-0) contains supplementary material, which is available to authorized users.

## Background

Rapid-onset obesity with hypothalamic dysfunction, hypoventilation, and autonomic dysregulation (ROHHAD) is a devastating pediatric disorder, the first sign of which is rapid-onset obesity (weight gain of 20–30 pounds over a 3–12 month period), occurring primarily between the ages of 2–7 years in a previously healthy child with normal neurocognitive development. Following the onset of obesity, other characteristic symptoms of hypothalamic dysfunction (including disordered water balance, growth hormone insufficiency, hyperprolactinemia, hypothyroidism, adrenal insufficiency and altered pubertal onset), alveolar hypoventilation, and autonomic dysregulation (including ophthalmologic manifestations, gastrointestinal dysmotility, thermal dysregulation with altered vasomotor tone, elevated pain sensing threshold, bradycardia, and neural crest tumors) emerge with a variable time course (often described as “unfolding” of the phenotype) [1]. If not diagnosed accurately and managed conservatively (i.e., imposing optimal artificial ventilation), worsening hypoventilation can result in impaired neurocognitive development and/or cardiorespiratory arrest [1]. Though initially described 50 years ago with a different name, to date there have been fewer than 100 cases of ROHHAD reported in the literature, and the disorder remains poorly understood [1–8]. The underlying etiology and the interrelationship among the various components of the ROHHAD phenotype are still unknown, though dysfunction of neural crest-derived cells and tissues is suspected.

Superficially, one might consider that ROHHAD bears some similarity to Prader-Willi syndrome (PWS), because both syndromes are marked by childhood obesity [9–11]. Caused by a lack of the paternal contribution of genes on chromosome 15q that are normally maternally-imprinted (i.e. silenced on the maternally-derived chromosome), PWS presents first with neonatal hypotonia, poor feeding and poor growth, followed by rapid weight gain in early childhood and compulsive food seeking behaviors in later childhood [9–12]*.* The other major symptoms of PWS are mild to moderate intellectual disability, characteristic behavioral disorders including compulsivity and a rigid cognitive style, growth hormone deficiency leading to short stature, hypogonadism, and a characteristic facial appearance with a thin upper lip, narrow nasal bridge, narrow forehead and almond-shaped eyes [10]. The presentation of PWS is highly variable, and can also include the following additional symptoms: sleep-disordered breathing, hypopigmentation, small hands and feet, strabismus, reduced visual acuity, scoliosis, hip dysplasia, osteopenia, seizures, decreased saliva volume, altered pain perception, altered temperature perception, high vomiting threshold, skin picking, and easy bruising [10].

In addition to childhood obesity, other shared features of ROHHAD and PWS are control of breathing abnormalities and signs of both hypothalamic dysfunction and autonomic dysregulation. Thus, non-experts may fail to recognize the differences, and the extremely rare ROHHAD patient could be misdiagnosed as having the more common disorder, PWS, which occurs in between 1 in 15,000 and 1 in 30,000 live births [10]. Indeed, ROHHAD patients are often tested for PWS when their physicians become aware of their obesity [1]. We therefore present here a detailed clinical comparison between ROHHAD and PWS, which is meant to help the pediatrician to clarify their differences and expedite the correct diagnosis of ROHHAD.

The observation of apparent similarities between the ROHHAD and PWS phenotypes has also given rise to the hypothesis that the two may share an underlying genetic origin. Since PWS patients lack the expression of multiple contiguous genes, one hypothesis is that coding mutations in just one of the PWS region genes can cause ROHHAD. Indeed, this is the case for another PWS-like syndrome, Schaaf-Yang syndrome, which is caused by loss of function mutations in the PWS region gene *MAGEL2* [13, 14]. To test if ROHHAD patients carry rare coding mutations in any of these genes, we analyzed the coding sequences of all the paternally-expressed PWS region genes in a cohort of seven ROHHAD probands.

## Methods

### Cohort

The seven ROHHAD patients included in the genetic analysis were described previously (see Discovery Cohort in reference [15]). All seven patients were clinically evaluated at The Center for Autonomic Medicine in Pediatrics (CAMP) at Ann & Robert H. Lurie Children’s Hospital of Chicago and the Stanley Manne Children’s Research Institute, which is a Center of Excellence for the study of ROHHAD. All seven met the diagnostic criteria for ROHHAD [1], displaying rapid-onset obesity after a period of normal development (mean age at onset: 4.4 years), hypothalamic dysfunction, hypoventilation, and autonomic dysregulation. All required artificial ventilation, and five of the seven had a benign tumor of neural crest origin.

### Prader-Willi syndrome diagnostic testing

The standard clinical diagnostic test for PWS is a DNA methylation-sensitive test that determines whether or not the paternal contribution of the Prader-Willi syndrome regions is both present and methylated appropriately. Normally, the maternal chromosome is imprinted (methylated) such that the genes in this region are not expressed. The paternal chromosome, on the other hand, is not imprinted, so the paternally-derived copies of the genes are normally expressed. PWS occurs when the paternal copy of the PWS region is either absent (due to a paternal deletion or maternal uniparental disomy of chromosome 15) or imprinted and silenced (due to a methylation error). A variety of methylation-based tests, including methylation-sensitive multiplex ligation-dependent probe amplification (MS-MLPA), can detect abnormalities consistent with a diagnosis of PWS. MS-MLPA was carried out in the Molecular Diagnostic Laboratory at the Alberta Children’s Hospital (Calgary, AB), as per standard procedures, for each of the seven ROHHAD probands.

### Sample collection and DNA extraction

Genomic DNA was isolated from peripheral blood samples, using a Puregene reagent kit (Qiagen).

### Next-generation sequencing (NGS) and analysis

Exome captures were done via the Agilent SureSelect capture kit V5 + UTRs. Massively parallel sequencing was performed on a SOLiD platform and sequences were aligned to the human reference genome (GRCh37) using Lifescope Genomic Analysis Software 2.5 (Life Technologies). Variants were called using the Genome Analysis Toolkit (GATK) Haplotype Caller (Version 3.3) [16], and annotated for filtration and prioritization using ANNOVAR [17]. We analyzed the eleven paternal-only expressed genes in the PWS region (Table 1). For the protein-coding genes, candidate mutations were identified as novel or rare (Minor Allele Frequency (MAF) < 0.005, according to the Exome Aggregation Consortium (ExAC [18])), non-synonymous, or splice site (within 2 bp of an exon) variants that were not within a segmental duplication. For the genes whose products are non-coding RNA, candidate mutations were any variant located within the boundaries of the gene (Table 1) with MAF < 0.005.

**Table 1:** Sequence Analysis of Paternal-Only Expressed Genes Within the Prader-Willi Syndrome Region (PWS Region)

PWS Region Gene	Region Analyzed^a^ (Ensembl GRCh37 release 89^b^)	Percent of Analyzed Bases Covered > 20× by NGS
*MKRN3*	chr15:23810929–23812453	100
*MAGEL2*	chr15:23889139–23892889	71 (100% by Sanger sequencing)
*NDN*	chr15:23931398–23932364	68 (Previously excluded [5])
*NPAP1*	chr15:24921014–24924485	91 (100% covered > 10×)^c^
*SNURF-SNRPN*	chr15:25219600-25219603	91 (100% covered > 10×)^c^
	chr15:25220504–25220656	
	chr15:25221451–25221563	
	chr15:25222023–25222176	
	chr15:25222924–25223063	
	chr15:25223339–25223465	
	chr15:25223553–25223591	
*SNORD107*	chr15: 25227140–25227215	100
*SNORD64*	chr15: 25230246–25230313	100
*SNORD109A*	chr15:25287120–25287187	100
*SNORD116* (cluster)	*See* Additional file 1	100
*SNORD115* (cluster)	*See* Additional file 1	98^d^
*SNORD109B*	chr15:25523489–25523556	100

^a^For the protein-coding genes, the coding region is listed; for the non-coding transcripts (snoRNA genes), the transcribed region is listed

^b^The coding region of *MAGEL2* is according to UCSC

^c^10× coverage allows for reasonably confident variant calling, and since 91% was covered at > 20×, and 9% was covered between 10×-20×, these genes were considered to be adequately covered by the NGS

^d^42/44 members of the cluster (all except the two shorter members, *SNORD115–45* and *SNORD115–47*; see Additional file 1) were completely covered at > 20×

### Sanger sequencing

We used the NCBI Primer Blast tool to design site-specific primers in order to amplify the entire coding region of *MAGEL2* (chr15: 23889139–23892889; GRCh37). Amplicons were purified using a spin purification protocol (Omega Biotek) and sequenced in both forward and reverse directions using the fluorescent dideoxy terminator method (Sanger method). Amplicon sequences were compared to the reference genome (GRCh37) using Mutation Surveyor (SoftGenetics) in order to identify any variant positions within the exon or canonical splice sites. As noted above, candidate mutations were identified as novel or rare (MAF < 0.005), non-synonymous variants.

## Results

### ROHHAD vs. PWS – Clinical comparison

Based on literature reviews and our own clinical observations and experience we compared the typical presentation of each condition. Table 2 lists the main symptoms of each condition, and which symptoms overlap between the two conditions (Table 2; bolded rows represent apparently overlapping symptoms).

**Table 2:** Clinical Comparison Between ROHHAD & Prader-Willi Syndrome Phenotypes

Feature	Present in ROHHAD	Present in PWS
Main Features of ROHHAD		
Normal neonatal development	Yes	No
**Rapid-onset obesity**	**Yes** *Over 3–12 months.*	**Yes** *Less steep trajectory.*
**Hypoventilation**	**Yes** *Universal and severe - All ROHHAD patients eventually require, at a minimum, artificial ventilation during sleep, with as many as half requiring ventilation 24 h per day. If adequate ventilatory support is not provided, many ROHHAD patients suffer a cardiorespiratory arrest* [1, 4, 5, 20–25]*.*	**Sometimes** *Not universal, though at times severe, with some instances of cardiorespiratory arrest reported* [33, 34]*.*
**Hypothalamic dysfunction**	**Yes**	**Yes**
Inability to maintain normal water balance	Yes	No
**Failed growth hormone stimulation test or growth hormone insufficiency**	**Sometimes** *Only rarely manifests as slowed growth rate or short stature.*	**Yes** *Growth hormone deficiency results in the characteristic short stature of PWS patients.*
Hyperprolactinemia	Yes	No
**Hypothyroidism**	**Sometimes**	**Sometimes**
**Adrenal insufficiency**	**Sometimes**	**Sometimes**
**Early or late puberty**	**Sometimes**	**Sometimes**
**Autonomic dysregulation**	**Yes**	**Yes**
Bradycardia	Sometimes *Typically temperature-related.*	No
**Gastrointestinal dysmotility**	**Yes**	**Sometimes**
Neural crest tumors (typically benign)	Yes *40–50% of cases*	No
**Ophthalmologic manifestations**	**Yes**	**Yes**
**Pain perception altered (elevated threshold)**	**Yes**	**Yes**
Sweating (profuse)	Yes	No
**Thermal dysregulation (reduced core temperature)**	**Yes** *Manifests most typically as hypothermia, despite a preference for light weight clothing regardless of ambient temperature.*	**Yes** *PWS patients are seen to have unstable temperatures, becoming hyper- or hypothermic easily in hot or cold environments and responding poorly to fevers, with very high temperatures occurring when ill, and sometimes without explanation. PWS patients also have a higher threshold for thermal sensing.*
Vasomotor tone (ice cold hands and feet)	Yes	No
Main Features of PWS		
Decreased fetal movement	No	Yes
Neonatal hypotonia	No	Yes
Neonatal feeding problems	No	Yes
Neonatal lethargy	No	Yes
Delayed motor skills	No	Yes
Hypogonadism	No	Yes
**Early or delayed puberty, stalled puberty**	**Sometimes**	**Yes**
Intellectual disability or developmental delay preceding obesity	No *Most ROHHAD patients have normal IQ. However, there are rare cases of developmental delay and one case of mild intellectual disability* [1]*. It has been hypothesized however, that these issues occur only after inadequate oxygenation due to undiagnosed or improperly managed hypoventilation* [8]*.*	Yes *Children with PWS are delayed in reaching cognitive, motor, and language milestones. Older PWS patients have IQ scores ranging from the high range for intellectual disability to the low range of normal, with most PWS patients showing mild intellectual disability. In addition, most PWS patients have learning disabilities.*
Autism spectrum disorder	No	Yes *Diagnosed in up to a quarter of PWS patients.*
**Maladaptive behavior (impulsive, compulsive, manipulative)**	**Sometimes** *It has been our experience that most ROHHAD patients, as long as their hypoventilation has been adequately managed, have no disordered behavior. However, there are case reports of severe behavioral issues including anxiety, depression, rage, aggressiveness, psychosis, and obsessive compulsive disorder* [1, 23, 25, 35–37]*. It has been hypothesized however, that these issues occur only after inadequate oxygenation due to undiagnosed or improperly managed hypoventilation, since these symptoms are not seen in patients who were diagnosed early and managed conservatively, and have not experienced a frank cardiopulmonary arrest* [8]	**Yes** *There is a characteristic disordered behavioral pattern among PWS patients that includes temper tantrums, stubbornness, rigidity, compulsiveness, and controlling or manipulative behaviors. Psychosis is seen in a small number of PWS patients (5–10%).*
**Eye abnormalities (strabismus)**	**Yes**	**Yes**
**Growth hormone deficiency**	**Sometimes**	**Yes**
Short stature	No	Yes
**Excessive weight gain independent of oral intake**	**Yes**	**Yes**
Hyperphagia-induced obesity	No *Initial weight gain is accompanied by only mild hyperphagia. But while a restricted diet can usually slow weight gain in ROHHAD, weight gain cannot be halted completely, and weight loss is exceedingly difficult to achieve.*	Yes *Weight gain initially occurs without noticeable hyperphagia, but then even greater weight gain occurs with extreme hyperphagia later in childhood.*
Excessive daytime sleepiness	No	Yes
Sleep abnormalities	No	Yes
**Central apnea**	**Rarely** *(hypoventilation is more characteristic)*	**Yes**
**Obstructive sleep apnea**	**Sometimes** *Initially, but resolves with intervention.*	**Yes**
Reduced activity	No	Yes
Higher threshold for thermal sensing	No	Yes
Decreased saliva flow	No	Yes
High vomiting threshold	No	Yes
**Scoliosis**	**Sometimes** *In ROHHAD, scoliosis is generally associated with thoracic neural crest tumors, whereas these tumors do not occur in PWS. However there is one reported case of a ROHHAD patient with scoliosis without a neural crest tumor* [38]*.*	**Yes** *Scoliosis is more common in PWS than in ROHHAD, and likely due to the PWS-related hypotonia.*
Additional Features of PWS		
Small hands/feet	No	Sometimes
Dysmorphic facial features	No	Sometimes
**Central hypothyroidism**	**Sometimes**	**Sometimes**
**Central adrenal insufficiency**	**Sometimes**	**Sometimes**
Hip dysplasia	No	Sometimes
Osteopenia and osteoporosis	No	Sometimes
Skin picking	No	Sometimes
Temperature instability	No	Sometimes
Hypopigmentation	No	Sometimes
**Seizures**	**Sometimes** *In ROHHAD, seizures are typically linked to episodes of hypoxemia.*	**Sometimes** *A subset of PWS patients suffers from a generalized seizure disorder of ranging severity (from febrile seizures to generalized epilepsy)* [39]*.*

‘Yes’ indicates that the symptom is a typical finding among patients

‘No’ indicates that the symptom is not typically seen among patients

‘Sometimes’ indicates that the symptom does recur among patients, but not so often as to be called ‘typical’

Bolded rows represent apparently overlapping symptoms

ROHHAD data are primarily taken from references [1, 5], and from our own clinical experience, with additional references as listed in the table

PWS data are primarily taken from references [9–11], and from our own clinical experience, with additional references as listed in this Table

### PWS diagnostic testing

PWS diagnostic testing by MS-MLPA excluded a genetic diagnosis of PWS in each of the seven ROHHAD probands.

### Mutation analysis of genes within PWS region

Eleven paternal-only expressed genes or gene clusters are located within the PWS deletion interval, of which five encode proteins and the other six are non-coding, and produce sets of small nucleolar (sno) RNAs (Table 1). These eleven genes were analyzed in the seven ROHHAD probands by next generation sequencing (NGS) and (where necessary) Sanger sequencing, in an effort to identify potentially disease-causing mutations. Nine of the eleven genes were well covered by NGS sequencing (Table 1). NGS analysis did not identify mutations with MAF < 0.005 in any of the PWS region genes in any of the seven ROHHAD patients.

Two genes in the PWS region were not well covered in the NGS analysis: *MAGEL2* (29% covered less than 20×, including a 574 bp region from chr15:23891203–23891777 not covered at all) and *NDN* (32% covered less than 20×, including a 173 bp region from chr15:23932085–23932258 not covered at all). *NDN* was previously excluded as a common candidate gene for ROHHAD by Sanger sequencing of the entire coding region in 10 ROHHAD probands [5], therefore we did not analyze this gene further by Sanger sequencing. As NGS coverage of *MAGEL2* was incomplete, we Sanger sequenced the entire coding region of the single-exon *MAGEL2* gene in each of our seven ROHHAD probands and did not identify any coding variants with MAF < 0.005.

## Discussion

The clinical comparison of ROHHAD and PWS provides a clear picture of the divergent and overlapping features of the two obesity disorders (Table 2). Considering divergent symptoms first, one of the most important differences is that ROHHAD patients are healthy up until the onset of obesity, typically over 2 years of age. In contrast, PWS patients are never described as normal and healthy, as they present soon after birth with hypotonia, feeding problems, and weak cry or inactivity. These neonatal symptoms are universal among PWS patients [19], so a normal first year of life in the child with later abrupt and extreme weight gain should trigger consideration of a ROHHAD diagnosis. PWS patients also display many additional symptoms later in life (intellectual, behavioural, and physical) that are never, or only very rarely, observed in ROHHAD (see Table 2).

Considering overlapping symptoms next, we see that all four of the acronym characteristics of ROHHAD (rapid-onset obesity, hypothalamic dysfunction, hypoventilation, and autonomic dysregulation) are also observed (at least sometimes) in PWS. However, a more detailed look at each of these apparently overlapping symptoms reveals important differences, as detailed in Table 2.

Hypoventilation is one such overlapping symptom that bears additional discussion. Though both ROHHAD and PWS present with breathing abnormalities, they do not present in exactly the same way. ROHHAD patients often present with obstructive sleep apnea (OSA) [1, 20], and OSA usually precedes central hypoventilation during sleep [20]. However, interventions aimed at treating OSA do not prevent the eventual development of central hypoventilation during sleep and, in a subset of cases, later wakefulness. All ROHHAD patients eventually require at a minimum artificial ventilation during sleep, with as many as half requiring support 24 h per day [1]. If adequate ventilatory support is not provided, many ROHHAD patients suffer a cardiorespiratory arrest [1, 4, 5, 20–25]. This severe hypoventilation is not always present at initial diagnosis but may only appear later, usually between 3 and 7 years of age [1, 20]. A comprehensive evaluation of responses to ventilatory challenges in ROHHAD patients suggests that their chemosensory deficit is mild compared to controls, with a diminished tidal volume and inspiratory drive response to hypoxic hypercapnia, and a lack of awareness of the physiologic challenge, being the only significant differences [26]. Thus, the underlying cause of the extreme hypoventilation observed in these patients remains to be explained.

“Sleep-disordered breathing” in PWS patients is common and usually includes the following: OSA, sleep-related hypoxemia, hypoventilation, and reduced ventilatory responses to hypoxia and hypercapnia, with heightened risk due to additional factors such as hypotonia and small nasopharynx [27]. However, nocturnal hypoventilation and increased central apneas are also reported among PWS patients [28–30]. Reduced ventilatory responses to hypoxia and hypercapnia (seen as a failure to increase minute ventilation) and aberrant response to hyperoxia (seen as an increase in minute ventilation, in contrast to the decrease seen in controls in the same study [31]), and abnormal arousal thresholds, are well documented in PWS patients and appear to be due to abnormal peripheral chemoreceptor activity and not related to obesity [28, 31, 32]. Hypoventilation in PWS is not as severe as in ROHHAD, since most PWS patients do not require artificial ventilatory support. However, deaths due to hypoventilation have been reported among PWS patients. In a series of 27 deceased PWS cases, three infants under 1 year old died from hypoventilation [33] and another study of PWS patients over age 15 reported ventilatory failure to be a common cause of death [34].

The universality and severity of hypoventilation in ROHHAD requires that ROHHAD be accurately distinguished from all other pediatric obesity phenotypes. When correctly diagnosed, ROHHAD patients can be vigilantly monitored by a respiratory physiologist, and then once signs of hypoventilation emerge, optimal oxygenation and ventilation can be maintained using artificial ventilation (mechanical ventilator or potentially phrenic nerve-diaphragm pacers). Since hypoventilation has been reported to cause death in PWS (though much more rarely than in ROHHAD), comprehensive respiratory physiology evaluation using comparable protocols to testing reported in ROHHAD may be prudent in PWS patients as well.

Though the two phenotypes implicate the same systems and functions (metabolic, respiratory, hypothalamic, endocrine), these systems seem to be affected in different ways in each of these obesity-related conditions. For example, though childhood obesity is one of the most striking characteristics of both ROHHAD and PWS, the two have different weight-gain trajectories, and different degrees of hyperphagia (see Table 2). Additionally, though OSA and hypoventilation are observed in both ROHHAD and PWS, responses to ventilatory challenges differ between them [26–32], suggesting that the underlying mechanisms of the observed hypoventilation diverge as well. Finally, the fact that different evidence of hypothalamic dysfunction and autonomic dysregulation is observed in each disorder would suggest that the underlying basis for hypothalamic and autonomic nervous system dysfunction differs as well. These important discrepancies in the specific symptoms in each disorder suggest that there are likely important differences in the underlying mechanisms behind them.

Recognition of the seeming overlap between ROHHAD and PWS led to the hypothesis that these syndromes may have similar etiological bases. Therefore, the genomic region known to be causative in PWS was examined in ROHHAD using standard PWS diagnostic testing and sequencing. We previously reported the results of a complete exome sequencing study of ROHHAD in which we did not identify causative mutations, ruling out rare protein-altering mutations across most of the genome [15]. In the present study, we have pointed out that the exome study did effectively rule out rare protein-altering mutations in most of the PWS region genes by providing at least 20× coverage across all protein-coding bases (see Table 1). Two genes (*NPAP1* and *SNURF-SNPRN*) had slightly reduced coverage, but since 91% was covered at > 20×, and the remaining 9% was covered between 10× and 20× (with 10× coverage still allowing for reasonably confident variant calling), these genes were considered to be adequately covered by the NGS. For the one gene where the NGS did not provide adequate coverage (*MAGEL2*), we have supplemented the exome data with Sanger sequencing data. Therefore, we have shown that ROHHAD is not caused by genetic mutations in any one of the genes in the PWS region. However, we have not yet examined the *expression levels* of any of these genes in ROHHAD patients, so it remains possible that the *expression* of one or more of these genes *is* altered in ROHHAD patients, possibly by epigenetic, transcriptional, or post-transcriptional misregulation. It is also possible that ROHHAD is caused by perturbations (at the level of genetic mutation or regulation of expression) in other genes in networks including one or more PWS region genes. Indeed, such an indirect relationship (as opposed to a direct overlap) between the underlying pathophysiologies of the two syndromes may be hypothesized, given the important differences between the phenotypes that we highlighted here.

## Conclusion

Despite superficial similarities, ROHHAD and PWS are distinct clinical conditions. Though the same systems and functions are involved in both phenotypes, they seem to be perturbed in different ways, and thus likely by different mechanisms. Many ROHHAD patients have had clinical testing that has ruled out a PWS diagnosis, and key features of PWS, especially neonatal hypotonia and failure to thrive, are not observed in ROHHAD patients. Nonetheless, pediatricians who may not be experts in either PWS or ROHHAD, may fail to recognize these differences immediately. If ROHHAD is not promptly diagnosed, then improper management leads to a threat of cardiorespiratory arrest or other morbidity. Thus, it is vital for the two conditions to be clearly separated in the minds of clinicians. To that end, we have provided a detailed clinical comparison of the two diseases, along with molecular evidence that an additional seven ROHHAD patients have neither classic PWS nor mutations in any PWS genes, as assessed in DNA from peripheral blood.

## Additional file

Additional file 1: Genomic Coordinates for Regions Analyzed for *SNORD115* and *SNORD116* Clusters (Ensembl GRCh37 Release 89). (XLSX 52 kb)

## Abbreviations

ANSD: Autonomic nervous system dysregulation
ExAC: Exome Aggregation Consortium
HD: Hypothalamic dysfunction
MAF: Minor Allele Frequency
MS-MLPA: Methylation-specific multiplex ligation-dependent probe amplification
NGS: Next-generation sequencing
OSA: Obstructive Sleep Apnea
PWS: Prader-Willi syndrome
ROHHAD: Rapid-onset obesity with hypothalamic dysfunction hypoventilation and autonomic dysregulation
snoRNA: Small nucleolar RNA
